# STCA-SNN: self-attention-based temporal-channel joint attention for spiking neural networks

**DOI:** 10.3389/fnins.2023.1261543

**Published:** 2023-11-10

**Authors:** Xiyan Wu, Yong Song, Ya Zhou, Yurong Jiang, Yashuo Bai, Xinyi Li, Xin Yang

**Affiliations:** School of Optics and Photonics, Beijing Institute of Technology, Beijing, China

**Keywords:** spiking neural networks, self-attention, temporal-channel, neuromorphic computing, event streams

## Abstract

Spiking Neural Networks (SNNs) have shown great promise in processing spatio-temporal information compared to Artificial Neural Networks (ANNs). However, there remains a performance gap between SNNs and ANNs, which impedes the practical application of SNNs. With intrinsic event-triggered property and temporal dynamics, SNNs have the potential to effectively extract spatio-temporal features from event streams. To leverage the temporal potential of SNNs, we propose a self-attention-based temporal-channel joint attention SNN (STCA-SNN) with end-to-end training, which infers attention weights along both temporal and channel dimensions concurrently. It models global temporal and channel information correlations with self-attention, enabling the network to learn ‘what’ and ‘when’ to attend simultaneously. Our experimental results show that STCA-SNNs achieve better performance on N-MNIST (99.67%), CIFAR10-DVS (81.6%), and N-Caltech 101 (80.88%) compared with the state-of-the-art SNNs. Meanwhile, our ablation study demonstrates that STCA-SNNs improve the accuracy of event stream classification tasks.

## 1. Introduction

As the representatives of mimicking the human brain at the neuronal level, Spiking Neural Networks (SNNs) have gained great attraction for the high biological plausibility, event-driven property, and high energy efficiency (Rieke et al., 1999; Gerstner et al., 2014; Bellec et al., 2018). Using time as an additional input dimension, SNNs record valuable information in a sparse manner and deliver information through spikes only when the membrane potential reaches the firing threshold (Mainen and Sejnowski, 1995). Inspired by biological visual processing mechanisms, Dynamic Vision Sensors (DVS) encode the time, location, and polarity of the brightness changes per pixel into event streams (Lichtsteiner et al., 2008; Posch et al., 2010). With its unique advantages of high event rate, high dynamic range, and fewer resource requirements (Gallego et al., 2020), DVS has broad application prospects in various visual tasks, such as autonomous driving (Cheng et al., 2019), high-speed object tracking (Rebecq et al., 2019), optical flow estimation (Ridwan and Cheng, 2017), and action recognition (Amir et al., 2017). Event-based vision is one of the typical advantage application scenarios of SNNs, providing a platform for demonstrating the capabilities of spiking neurons to process information with spatio-temporal dynamics.

Although the intrinsic time-dependent neuron dynamics endows SNNs with the ability to process spatio-temporal information, there remains a performance gap between SNNs and ANNs. Recently, ANNs’ modules (Hu et al., 2021; Yang et al., 2021; Yao et al., 2021, 2023c) have been integrated into SNNs to improve the performance of SNNs. CSNN (Xu et al., 2018) first validated the application of convolution structure on SNNs, promoting the development of SNNs. Convolution-based SNNs share weights across both temporal and spatial dimensions, following the assumption of spatio-temporal invariance (Huang et al., 2022). This approach can be regarded as a local way of information extraction since convolutional operations can only process a local neighborhood at a time, either in space or time. However, when dealing with sequential data like event streams, capturing long-distance dependencies is of central importance to modeling complex temporal dynamics. Non-local operations (Wang et al., 2018) provided a solution as a building block by computing the response at a position as a weighted sum of the features at all positions. The range of positions can span across space, time, or spacetime, allowing non-local operators to achieve remarkable success in vision attention.

**Figure 1:**
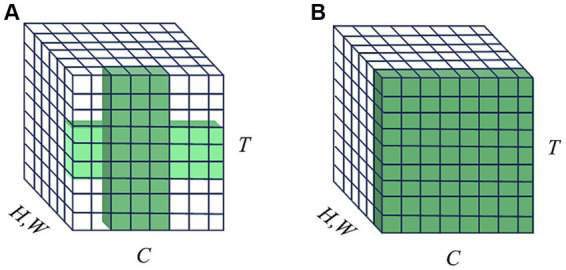
Illustration of receptive fields on channel and temporal domains. *T* means the temporal domain, *C* means the channel domain, and *H*, *W* represent the spatial domain. **(A)** TCJA-SNN utilizes two local attention mechanisms with 1-D convolution along temporal-wise and channel-wise, respectively, then fuse them, forming a cross-shaped receptive field. **(B)** STCA-SNN uses self-attention operation to establish temporal-wise and channel-wise correlations, forming a global receptive field.

The attention mechanism is inspired by the human ability to selectively find prominent areas in complex scenes (Itti et al., 1998). A popular research direction is to present attention as a lightweight auxiliary unit to improve the representation power of the basic model. In the ANNs domain, Ba et al. (2014) first introduced the term “visual attention” for image classification tasks, utilizing attention to identify relevant regions and locations within the input image. This approach also reduces the computational complexity of the proposed model regarding the size of the input image. SENet (Hu et al., 2018) was introduced to reweight the channel-wise responses of the convolutional features, determining “what” to pay attention to. CBAM (Woo et al., 2018) inferred attention maps sequentially along channel-wise and spatial dimensions for refining the input feature, determining “what” and “where” to pay attention to concurrently. In the SNNs domain, TA-SNN (Yao et al., 2021) first extended the channel-wise attention concept to temporal-wise attention and integrated it into SNNs to determine ‘when’ to pay attention. MA-SNN (Yao et al., 2023c) extended CBAM to SNNs and proposed a multi-dimensional attention module along temporal-wise, channel-wise, and spatial-wise separately or simultaneously. Recently, TCJA-SNN (Zhu et al., 2022) cooperated temporal-wise and channel-wise attention correlations using the 1-D convolution operation to present the correlation between time-steps and channels. However, the receptive field of TCJA-SNN is a local cross shape that is restricted by its convolution kernels, shown in Figure 1A. Thus long-range dependencies can only be captured when 1-D convolution operation is repeated, which makes multi-hop dependency modeling difficult. On the other hand, self-attention, another vital feature of the human biological system, possesses the ability to capture feature dependencies effectively as an additional non-local operator alongside SE and CBAM. It has sparked a significant wave of interest and achieved remarkable success in various tasks (Vaswani et al., 2017; Dosovitskiy et al., 2020; Liu et al., 2021). Intuitively, there is a compelling interest in investigating the application of self-attention in SNNs to advance deep learning, when considering the biological characteristics of both mechanisms (Yao et al., 2023a,b; Zhou C. et al., 2023; Zhou Z. et al., 2023).

To address the local spatio-temporal receptive field limitation of TCJA, we first adopt self-attention, a non-local operation, to model global temporal and channel information correlations. The self-attention module we employed can capture the global spatio-temporal receptive field, as shown in Figure 1B, allowing for the direct long-range dependencies modeling, which is the highlight of our work. We propose a plug-and-play Self-attention-based Temporal-Channel joint Attention (STCA) module for SNNs with end-to-end training. The STCA-SNNs can learn to focus on different features of the input at each time-step. In other words, the STCA-SNNs can learn ‘when’ and ‘what’ to attend concurrently, enhancing the ability of the SNNs to process temporal information. We evaluated the effectiveness of STCA-SNNs across different architectures on three benchmark event stream classification datasets: N-MNIST, CIFAR10-DVS, and N-Caltech 101. Our detailed experiments show that STCA-SNNs achieve competitive accuracy with existing state-of-the-art SNNs.

The main contributions of our work are summarized as follows:

1. We propose STCA-SNNs for event streams that can undertake end-to-end training and inference tasks.
2. The plug-and-play STCA module models global temporal and channel correlations with self-attention, allowing the network to learn ‘when’ and ‘what’ to attend simultaneously. This enhances the ability of SNNs to process temporal information.
3. We evaluate the performance of STCA-SNNs on three benchmark event stream classification datasets, N-MNIST, CIFAR10DVS, and N-Caltech 101. Our experimental results demonstrate that STCA-SNNs achieve competitive accuracy compared to existing state-of-the-art SNNs.

## 2. Related work

### 2.1. Attention in SNNs

Spiking neural networks benefit from biological plausibility and continuously pursue the combination with brain mechanisms. The attention mechanism draws inspiration from the human ability to selectively identify salient regions within complex scenes and has gained remarkable success in deep learning by allocating attention weights preferentially to the most informative input components. A popular research direction is to present attention as an auxiliary module that can be easily integrated with existing architectures to boost the representation power of the basic model (Hu et al., 2018; Woo et al., 2018; Guo et al., 2022; Li et al., 2022). Yao et al. (2021) first suggested using an extra plug-and-play temporal-wise attention module for SNNs to bypass a few unnecessary input timesteps. Then they proposed a multi-dimensional attention module along temporal-wise, channel-wise, and spatial-wise separately or simultaneously to optimize membrane potentials, which in turn regulate the spiking response (Yao et al., 2023c). STSC-SNN (Yu et al., 2022) employed temporal convolution and attention mechanisms to improve spatio-temporal receptive fields of synaptic connections. SCTFA-SNN (Cai et al., 2023) computed channel-wise and spatial-wise attention separately to optimize membrane potentials along the temporal dimension. Yao et al. (2023a,b) recently proposed an advanced spatial attention module to harness SNNs’ redundancy, which can adaptively optimize their membrane potential distribution by a pair of individual spatial attention sub-modules. TCJA-SNN (Zhu et al., 2022) cooperated temporal-wise joint channel-wise attention correlations using 1-D convolution operation. However, the temporal-channel receptive field of TCJA is a local cross shape that is restricted by its convolution kernels, requiring multiple repeated computations to establish long-range dependencies of features. Therefore, it is computationally inefficient and makes multi-hop dependency modeling difficult.

Among the attention mechanisms, self-attention, as another important feature of the human biological system, possesses the ability to capture feature dependencies. Originally developed for natural language processing (Vaswani et al., 2017), self-attention has been extended to computer vision, where it has achieved significant success in various applications. The self-attention module can also be considered a building block of CNN architectures, which are known for their limited scalability when it comes to large receptive fields (Han et al., 2022). In contrast to the progressive behavior of convolution operation, self-attention can capture long-range dependencies directly by computing interactions between any two positions, regardless of their positional distance. Moreover, it is commonly integrated into the top of the networks to enhance high-level semantic features for vision tasks. Recently, an emerging research direction is to explore the biological characteristics associated with the fusion of self-attention and SNNs (Yao et al., 2023a,b; Zhou C. et al., 2023; Zhou Z. et al., 2023). These efforts primarily revolve around optimizing the computation of self-attention within SNNs by circumventing multiplicative operations, leading to performance degradation. Diverging from these studies, our primary goal is to explore how self-attention can enhance the spatio-temporal information processing capabilities of SNNs.

### 2.2. Learning algorithms for SNNs

Existing SNN training methods can be roughly divided into three categories: 1) the biologically plausible method, 2) the conversion method, and 3) the gradient-based direct training method. The first one is based on biological plausible local learning rules, like spike timing dependent plasticity (STDP) (Diehl and Cook, 2015; Kheradpisheh et al., 2018) and ReSuMe (Ponulak and Kasinski, 2010), but achieving high performance for deep networks is challenging. The conversion method offers an alternative way to obtain high-performance SNNs by converting a well-trained ANN and mapping its parameters to an SNN with an equivalent architecture, where the firing rate of the SNN acts as ReLU activation (Cao et al., 2015; Rueckauer et al., 2017; Sengupta et al., 2019; Ding et al., 2021; Bu et al., 2022; Wu et al., 2023). Moreover, some works explored post-conversion fine-tuning of converted SNNs to reduce latency and increase accuracy (Rathi et al., 2020; Rathi and Roy, 2021; Wu et al., 2021). However, this method is not suitable for neuromorphic datasets. The gradient-based direct training methods primarily include voltage gradient-based (Zhang et al., 2020), timing gradient-based (Zhang et al., 2021), and activation gradient-based approaches. Among them, the activation gradient-based method demonstrates notable effectiveness when performing challenging tasks. This approach uses surrogate gradients to address the non-differentiable spike activity issue, allowing for error back-propagation through time (BPTT) to interface with gradient descent directly on SNNs for end-to-end training (Neftci et al., 2019; Wu et al., 2019; Yang et al., 2021; Zenke and Vogels, 2021). These efforts have shown strong potential in achieving high performance by exploiting spatio-temporal information. However, further research is required to determine how to make better use of spatio-temporal data and how to efficiently extract spatio-temporal features. This is what we want to contribute.

## 3. Materials and methods

In this section, we first present the representation of event streams and the adopted spiking neuron model and later propose our STCA module based on this neuron model. Finally, we introduce the training method adopted in this paper.

### 3.1. Representation of event streams

An event, e, encodes three pieces of information: the pixel location (*x*, *y*) of the event, the timestamp *t*′ recording the time when the event is triggered, and the polarity of each single event *p* ∈ {−1, +1} reflecting an increase or decrease of brightness via +1/−1. Formally, a set of events at the timestamp *t*′can be defined as:


(1) $$E_{t^{\prime }}={\left\{\left[x_{k},\;y_{k},\;t^{\prime },\;p_{k}\right]\right\}}_{k=1}^{N}$$

Assume the spatial resolution is *h* × *w*, the event set equals to the spike pattern tensor *S_t′_*∈R^2 × h × w^ at the timestamp *t*′. However, processing these events one by one can be inefficient due to the limited amount of information contained in a single event. We follow the frame-based representation in SpikingJelly (Fang et al., 2020) that transforms event streams into high-rate frame sequences during preprocessing. Each frame includes many blank (zero) areas, and SNNs can skip the computation of the zero areas in each input frame (Roy et al., 2019), improving overall efficiency.

### 3.2. Spiking neural models

Spiking neuron in SNNs integrates synaptic inputs from the previous layer and the residual membrane potential into the latest membrane potential. The Parametric Leaky integrate-and-fire (PLIF) model can learn the synaptic weight and membrane time constant simultaneously, which can enhance the learning capabilities of SNNs (Fang et al., 2021). The subthreshold dynamics of the PLIF neuron is defined as:


(2) $$\tau \frac{dV\left(t\right)}{dt}=-\left(V\left(t\right)-V_{\mathrm{rest}}\right)+X\left(t\right)$$

where *V* (*t*) indicates the membrane potential of the neuron at time *t*, *τ* is the membrane time constant that controls the decay of *V* (*t*), *X* (*t*) is the input collected from the presynaptic neurons and *V_rest_* is the resting potential. When the membrane potential *V* (*t*) exceeds the neuron threshold at time *t*, the neuron will emit a spike, and then the membrane potential goes back to a reset value *V_rest_*. We set *V_rest_* = *V_reset_* = 0. The iterative representation of the PLIF model can be described as follows:


(3) $$\left\{ \begin{array}{c} H^{t,l}=V^{t-1,l}+\frac{1}{\tau }\left(-\left(V^{t-1,l}-V_{\mathrm{reset}}\right)+X^{t,l}\right)\\ S^{t,l}=\Theta \left(H^{t,l}-V_{th}\right)\\ V^{t,l}=\left(1-S^{t,l}\right)H^{t,l}+V_{re\mathrm{set}}S^{t,l} \end{array}\right.$$

where superscripts *t* and *l* indicate the time step and layer index. To avoid confusion, we use *H^t,l^* and *V^t,l^* to represent the membrane potential after neuronal dynamics and after the trigger of a spike in layer *l* at time-step *t*, respectively*. V_th_* is the firing threshold. *S^t,l^* is determined by $\Theta \left(x\right)$ , the Heaviside step function that outputs 1 if *x* ≥ 0 or 0 otherwise. The time constant *τ =* 1*/k*(*a*), *k*(*a*) is a sigmoid function 1/(1 + exp(−*a*)) with a trainable parameter *a*.

### 3.3. Self-attention-based temporal-channel joint attention module

**Figure 2:**
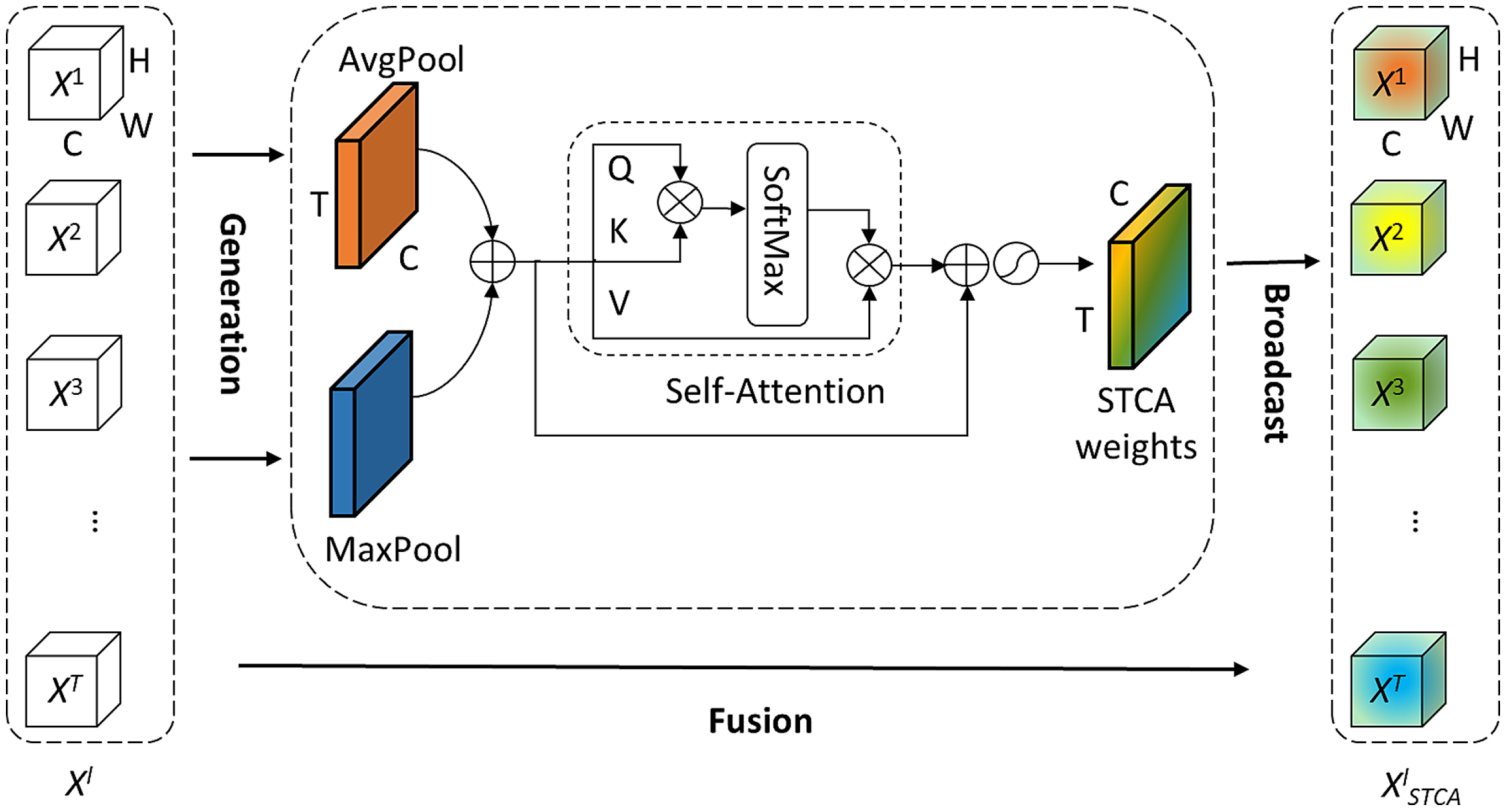
Diagram of the STCA module. The STCA module first aggregates spatial information by average-pooling and max-pooling then merges them and feeds it into a self-attention block to establish the correlations in both temporal and channel dimensions.

The processing of temporal information in SNNs is generally attributed to spiking neurons because their dynamics naturally depend on the temporal dimension. However, the LIF neuron and its variants including the PLIF neuron, only sustain very weak temporal linkages. Additionally, event streams are inherently time-dependent therefore, it is necessary to establish spatial–temporal correlations to improve data utilization. The focus of this work is to model temporal-wise and channel-wise attention correlations globally by adopting a self-attention mechanism. We present our idea of attention with a pluggable module termed the Self-attention-based Temporal-Channel joint Attention (STCA), which is depicted in Figure 2.

Formally, we collect intermediate the spatial feature of *l*-th layer at all time-steps *X^l^* = [· · ·, *X^t,l^*, · · ·]∈ R^T × C × H × W^ as the input of STCA module, where *T* is time-step, *C* denotes channels, *H* and *W* are height and width of the feature, respectively. The spatial feature *X^t,l^* can be extracted from the original input *S^t,l^*:


(4) $$X^{t,l}=BN\left(\mathrm{Conv}\left(W^{l},S^{t,l-1}\right)\right)$$

where BN (·) and Conv (·) mean the batch normalization and convolutional operation, *W^l^* is the weight matrix, *S*^*t*, *l-1*^ (*l* ≠ 1) is a spike tensor that only contains 0 and 1, and $X^{t,l}\in R^{C_{l}\times H\times W_{l}}$ . To simplify the notation, bias terms are omitted. BN is a default operation following the Conv, we also omit it in the rest of this paper. Since each spatial feature *X^t,l^* in *X^l^* is time-dependent, our idea of attention is to utilize the temporal correlation of these features. It is well known that each channel of feature maps corresponds to a specific visual pattern. Our STCA module aims to determine ‘when’ to attend to ‘what’ are semantic attributes of the given input. For efficiency, STCA only focuses on temporal and channel modeling, the spatial information of the feature is aggregated by using both avg-pooling and max-pooling operations as follows:


(5) $$R^{l}=\mathrm{AvgPool}\left(X^{l}\right)+\mathrm{MaxPool}\left(X^{l}\right)$$

where AvgPool (·) and MaxPool (·) represent the outputs of the avg-pooling and max-pooling layer respectively, *R^l^*∈R^T × C^. The generated different temporal-channel context descriptors, avg-pooled features and max-pooled features, are merged and then fed into a self-attention (SA) block. We follow the convention (Wang et al., 2018) to formulate the SA block, where the input feature in layer *l* is *R^l^*∈R^T × C^, and the output feature is generated as:


(6) $$a_{i}^{l}=\frac{1}{C\left(r_{i}\right)}\underset{\forall j}{\sum }f\left(r_{i},r_{j}\right)g\left(r_{j}\right)$$

where *r_i_*∈R ^1 × C^ and *a_i_*∈R^1 × C^ indicate the *i^th^* position of the input feature *R^l^* and output feature *A^l^*, respectively. Subscript *j* is the index that enumerates all positions along the temporal domain, i.e., *i*, *j*∈[1,2,…, T], and a pairwise function *f* (·) computes a representing relationship between *i* and all *j.* The function *g* (·) computes a representation of the input signal at time-step *j*, and the response is normalized by a factor *C* (*r_i_*). We use a simple extension of the Gaussian function to compute the similarity in an embedding space, and the function *f* (·) can be formulated as:


(7) $$f\left(r_{i},r_{j}\right)=e^{\theta \left(r_{i}\right)\emptyset {\left(r_{j}\right)}^{T}}$$

where *θ* (·) and *ϕ* (·) can be any embedding layers. If we consider the *θ* (·), *ϕ* (·), *g* (·) in the form of linear embedding: *θ* (*R^l^*) = *R^l^W_θ_*, *ϕ* (*R^l^*) = *R^l^W_ϕ_*, *g* (*R^l^*) = *R^l^W_g_*, where *W_θ_*∈ $R^{C\times C_{k}}$ , *W_ϕ_* ∈ $R^{C\times C_{k}}$ *, W_g_* ∈ $R^{C\times C_{k}}$ , and set the normalization factor as $C\left(r_{i}\right)=\underset{\forall j}{\sum }f\left(r_{i},r_{j}\right)$ , the Eq. 6 can be rewritten as:


(8) $$a_{i}^{l}=\frac{e^{r_{i}w_{\theta ,i}w_{\emptyset ,j}^{T}r_{j}^{T}}}{\sum_{j}e^{r_{i}w_{\theta ,i}w_{\emptyset ,j}^{T}r_{j}^{T}}}r_{j}w_{g,j}$$

where ***w****_θ,i_*∈R^C × 1^ is the *i^th^* row of the weight matrix *W_θ_*. For a given index *i*, $\frac{1}{C\left(r_{i}\right)}f\left(r_{i},r_{j}\right)$ becomes the softmax output along the dimension *j.* The formulation can be future rewritten as:


(9) $$A^{l}=\mathrm{softmax}\left(R^{l}W_{\theta }W_{\emptyset }^{T}R^{l}\right)g\left(R^{l}\right)$$

where *A^l^*∈R^T × C^ is the output feature of the same size as *R^l^*. Given the query, key, and value representations:


(10) $$Q=R^{l}W^{Q},K=R^{l}W^{K},V=R^{l}W^{V}$$

Once $W^{Q}=W_{\theta }$ , $W^{K}=W_{\phi }$ , $W^{V}=W_{g}$ , *W^Q^*∈R^C × C^, *W^K^*∈R^C × C^, and *W^V^*∈R^C × C^, Eq. 9 can be formulated as:


(11) $$A^{l}=\mathrm{softmax}\left(QK^{T}\right)V$$

In this way, the SA block is constructed. Then we employ a residual connection around the SA block. Finally, the attention process of STCA can be formulated as:


(12) $$X_{\mathrm{STCA}}^{l}=f⊙X^{l}$$

where *f* = *σ*(*R^l^ + A^l^*) ∈ *R*^T × C^ is the weight vector of STCA, ⊙ is element-wise multiplication, *σ* is the sigmoid function, and *X^l^_STCA_*∈R^T × C × H × W^ denotes the feature extracted by the STCA module along temporal and channel dimensions.

### 3.4. Training

We integrate the STCA module into networks and utilize the BPTT method to train SNNs. Since the process of neuron firing is non-differentiable, we use the derived ATan surrogate function $\sigma^{\prime }\left(x\right)=\alpha /2{\left(1+\pi \alpha x/2\right)}^{2}\text{.}$ For a given input with label *n*, the neuron that represents class *n* has the highest excitatory level while other neurons remain silent. So the target output is defined by *Y* = [*y^t, i^*] with *y^t, i^* = 1 for *i* = *n*, and *y^t, i^* = 0 for *i* ≠ *n*. Then the loss function is described by the spike mean squared error:


(13) $$L={\left\|y^{i}-\frac{1}{T}\overset{T}{\underset{t=1}{\sum }}o^{t,i}\right\|}^{2}$$

where *O =* [*o^t, i^*] is the average spiking events of neurons under the voting strategy.

## 4. Experiments

### 4.1. Experimental setup

#### 4.1.1. Implementation details

We implement our experiments with the Pytorch package and SpikingJelly framework. All experiments were conducted using the BPTT learning algorithm on 4 NVIDIA RTX 2080 Ti GPUs. We utilized the Adam optimizer (Kingma and Ba, 2015) to accelerate the training process and implemented some standard training techniques of deep learning such as batch normalization and dropout. The corresponding hyper-parameters and SNN hyper-parameters are shown in Table 1. We verify our method on the following DVS benchmarks:

**Table 1:** Hyper-parameter setting.

Hyperparameter	N-MNIST	CIFAR10-DVS	N-Caltech 101
Max Epoch	500	1,000	500
Automatic mixed precision	x	x	✓
Batch size	64	32	8
Learning rate	1e-3	1e-3	1e-3
Time step	10	10	14
*V_th_*	1.0	1.0	1.0
*τ_0_*	2.0	2.0	2.0
head	4	4	4

CIFAR10-DVS contains 10 K DVS images of 10 classes recorded with the dynamic vision sensor from the original static CIFAR10 dataset. We apply a 9: 1 train-valid split (i.e., 9 k training images and 1 k validation images). The resolution is 128 × 128, we resize all of them to 48 × 48 in our training and we integrate the event data into 10 frames per sample (Li et al., 2017).

N-Caltech 101 dataset contains 8,831 DVS images converted from the original version of Caltech 101 with a slight change in object classes to avoid confusion. The N-Caltech 101 consists of 100 object classes plus one background class. Similarly, we apply the 9: 1 train-test split as CIFAR10-DVS. We use the SpikingJelly (Fang et al., 2020) package to process the data and integrate them into 14 frames per sample (Orchard et al., 2015).

The neuromorphic MNIST dataset is a converted dataset from the original static MNIST dataset (Orchard et al., 2015). It contains 50 K training images and 10 K validation images. We integrate the event data into 10 frames per sample using SpikingJelly (Fang et al., 2020) package.

#### 4.1.2. Networks

The network structures with STCA for different datasets are provided in Table 2 and the network architectures we use have been proven to perform quite well on each dataset. Specifically, for the CIFAR10-DVS dataset, we adopt a VGG11-like architecture. To mitigate the apparent overfitting on the CIFAR10-DVS dataset, we adopt the neuromorphic data augmentation, including horizontal Flipping and Mixup in each frame, which is also used in Zhu et al. (2022) for training the same dataset. For the N-Caltech 101 dataset, we adopt the same architecture with Zhu et al. (2022) and N-MNIST refers to PLIF Fang et al. (2021). The voting layers are implemented using average pooling for classification robustness.

**Table 2:** The network structures with STCA for different datasets.

Dataset	Network structure
N-MNIST	Input-128C3-Neuron-MP2-128C3-Neuron-STCA-MP2-0.5DP-2048FC-Neuron-0.5DP-100FC-Neuron-Voting
CIFAR10-DVS	Input-64C3-Neuron-128C3-Neuron-AP2-256C3-Neuron-256C3-Neuron-STCA-AP2-512C3-Neuron -512C3-Neuron-STCA-AP2-512C3-Neuron-512C3-Neuron-AP2-10FC-Neuron
N-Caltech 101	64C3-Neuron-MP2-128C3-Neuron-MP2-256C3-Neuron-STCA-MP2-256C3-Neuron-STCA-MP2-512C3-Neuron-0.8DP-1024FC-Neuron-0.5DP-101FC-Neuron

*x*C*y*/MP*y*/AP*y* denotes the Conv2D/MaxPooling/Avgpooling layer with output channel = *x*, and kernel size = *y* × *y* ,  *n* FC denotes the fully connected layer with output feature = *n*, MP*y* is the spiking dropout layer with dropout ratio *m*. BN follows behind all *x*C*y*.

### 4.2. Comparison with existing state-of-the-art works

Table 3 displays the accuracy performance of the proposed STCA-SNNs compared to other competing methods on three neuromorphic datasets, N-MNIST, CIFAR10-DVS, and N-Caltech 101. We mainly include direct training results of SNNs with signal transmission via binary spike. Among them, some works (Wu et al., 2019; Yao et al., 2021) replace binary spikes with floating-point spikes and maintain the same forward pipeline as SNNs to obtain enhanced classification accuracy. STCA-SNNs achieve better performance than existing state-of-the-art SNNs on all datasets. We first compare our method on the CIFAR10-DVS dataset. We continue to utilize MSE the loss function and the same network architecture as TCJA-SNN (Zhu et al., 2022) and STSC-SNN (Yu et al., 2022) to preserve the consistency of this work, and our method reaches 81.6% top-1 accuracy, improving the accuracy by 0.9% over TCJA-SNN (Zhu et al., 2022). We also compare our method on N-Caltech 101dataset. Under the same condition as TCJA-SNN (Zhu et al., 2022) with MSE the loss function, we get a 2.38% increase over it and outperform the comparable result. Finally, we test our algorithm on the N-MNIST dataset. As shown in Table 3, most comparison works get over 99% accuracy. We use the same architecture as PLIF. Our STCA-SNN reaches the best accuracy of 99.67%.

**Table 3:** Accuracy performance comparison between the proposed method and the SOTA methods on different datasets.

Method	Binary spikes	N-MNIST		CIFAR10-DVS		N-Caltech 101	
		T	Acc. (%)	T	Acc. (%)	T	Acc. (%)
tdBN (Yang et al., 2021)	✓	–	–	10	67.8	–	–
Rollout (Kugele et al., 2020)	✓	32	99.57	48	66.97	–	–
LIAF-Net (Wu et al., 2019)	x	20	99.13	10	70.4	–	–
ConvSNN (Samadzadeh et al., 2023)	✓	-	99.6	-	69.2	–	–
PLIF (Fang et al., 2021)	✓	10	99.61	20	74.80	–	–
TA-SNN (Yao et al., 2021)	x	–	–	10	72.0	–	–
SALT (Kim and Panda, 2021)	✓	–	–	20	67.1	20	55.0
STSC-SNN (Yu et al., 2022)	✓	10	99.64	10	81.4^a^	–	–
TCJA-SNN (Zhu et al., 2022)	✓	–	–	10	80.7^a^	14	78.5
This work	✓	10	99.67	10	81.6^a^	14	80.88

a With data augmentation.

### 4.3. Ablation study

#### 4.3.1. Ablation study

**Figure 3:**
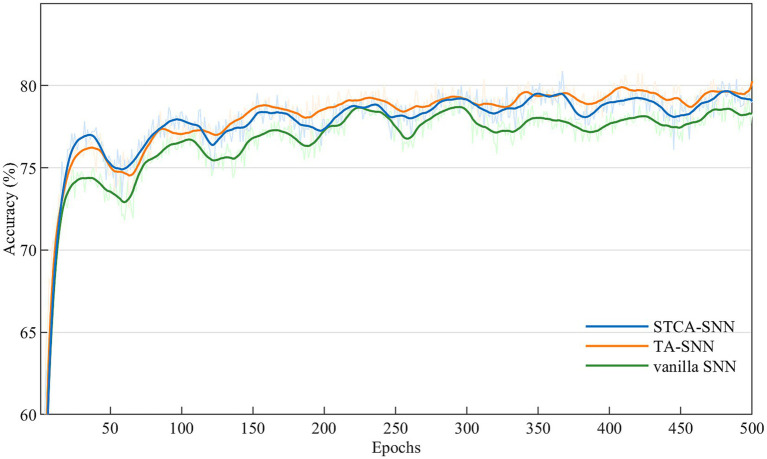
Convergence of compared SNN methods on N-Caltech101 dataset.

We performed ablation experiments based on the PLIF neuron model to evaluate the effectiveness of the STCA module. For each dataset, we trained three types of SNNs: STCA-SNNs, TA-SNNs with temporal-wise attention module (Yao et al., 2023c), and vanilla SNNs (PLIF-SNN) without any attention module. The SE attention employed by TA-SNNs in the temporal dimension and the Self-attention employed in this work are both non-local operators, thus, we compared the performance of these two classic non-local operators under the same experiment conditions. We followed the learning process described in section 4.1 for all ablation experiments, and the attention locations were identical for both TA-SNNs and STCA-SNNs. Table 4 shows that all STCA-SNNs outperformed vanilla SNNs on three event stream classification datasets, suggesting that the benefits of the STCA module are not limited to a specific dataset or architecture. Furthermore, Figure 3 illustrates the accuracy performance trend of vanilla SNN, TA-SNN, and our proposed STCA-SNN over 1,000 epochs on the N-Caltech101 dataset. As the training epoch increased, our proposed STCA-SNN demonstrated comparable performance with TA-SNN. This indicates that our STCA module can enhance the representation ability of SNNs.

**Table 4:** Accuracy of vanilla SNN, TA-SNN, and STCA-SNN models on different datasets.

Model	N-MNIST	CIFAR10-DVS	N-Caltech 101
Vanilla SNN	99.64	80.7	79.40
TA-SNN	99.64	81.3	80.76
STCA-SNN	99.67	81.6	80.88

#### 4.3.2. Discuss of pooling operations

**Figure 4:**
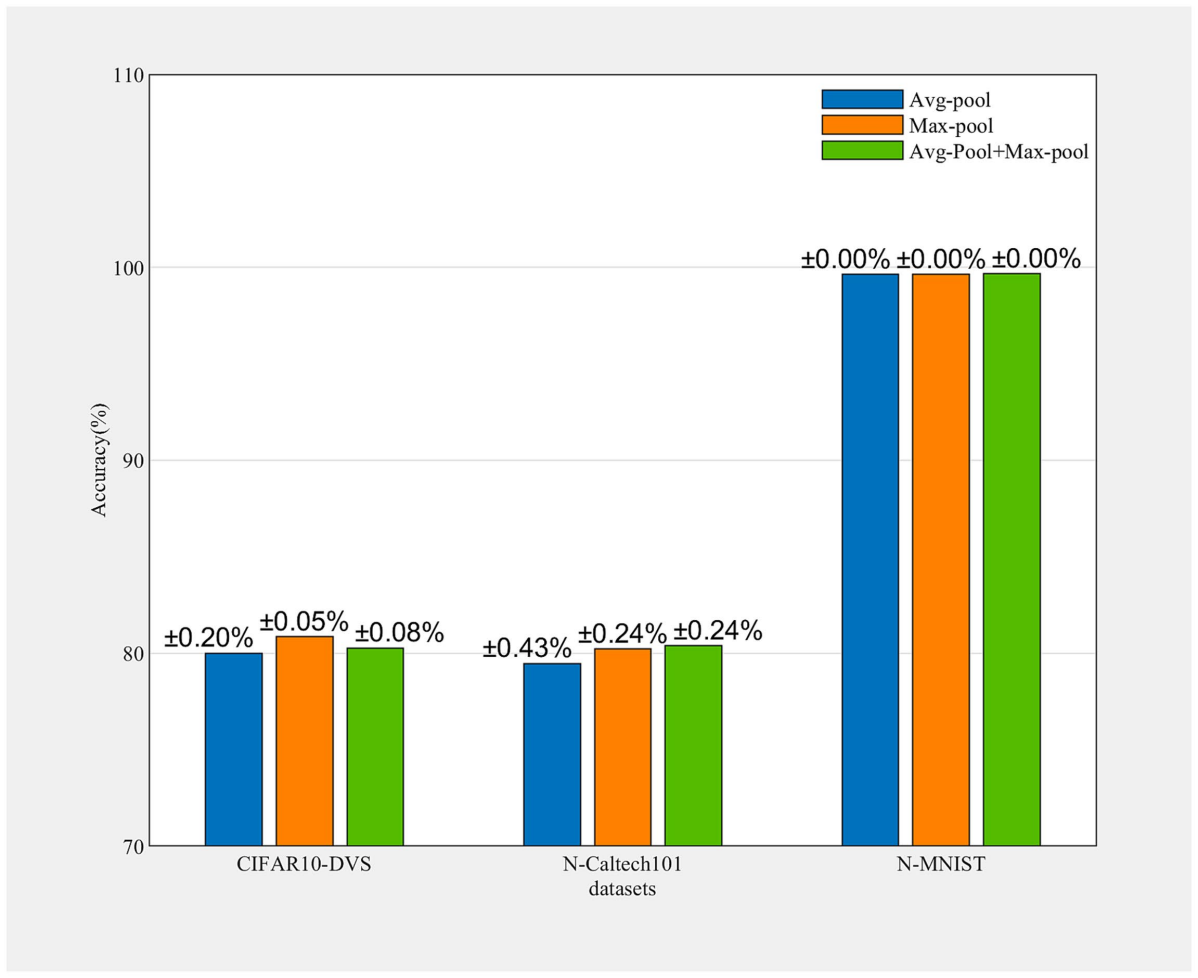
Accuracy of different datasets obtained by avg-pooling, max-pooling, and a combination of both. Each experiment is run 3 times.

To investigate the influence of the avg-pooling and max-pooling operation, we conducted several ablation studies. As is well known, avg-pooling can capture the degree information of target objects, while max-pooling can extract discriminative features of objects. As shown in Figure 4, the max-pooling operation contributes significantly to performance enhancement. Each experiment is run 3 times. Notably, the fusion of both pooling operations exhibits improved performance across all datasets examined, which means avg-pooling encoded global information can effectively compensate for the discriminative information encoded by max-pooling.

## 5. Conclusion

In this work, we propose the STCA-SNNs to enhance the temporal information processing capabilities of SNNs. The STCA module captures temporal dependencies across channels globally using self-attention, enabling the network to learn ‘when’ to attend to ‘what’. We verified the performance of STCA-SNNs on various neuromorphic datasets across different architectures. The experimental results show that STCA-SNNs achieve competitive accuracy on N-MNIST, CIFAR10-DVS, and N-Caltech 101 datasets.

## Data availability statement

The original contributions presented in the study are included in the article/supplementary material, further inquiries can be directed to the corresponding authors.

## Author contributions

XW: Conceptualization, Investigation, Methodology, Software, Visualization, Writing – original draft. YS: Funding acquisition, Supervision, Writing – review & editing. YZ: Supervision, Writing – review & editing. YJ: Supervision, Writing – review & editing. YB: Formal analysis, Validation, Writing – review & editing. XL: Formal analysis, Software, Validation, Visualization, Writing – review & editing. XY: Writing – review & editing.
